# Genome Signatures, Self-Organizing Maps and Higher Order Phylogenies: A Parametric Analysis

**Published:** 2007-09-17

**Authors:** Derek Gatherer

**Affiliations:** MRC Virology Unit, Institute of Virology. Church Street, Glasgow G11 5JR, UK

**Keywords:** Genome Signature, Self-Organizing Map, Viruses, Phylogeny, Jack-Knife Method, Microarray, Metagenomics, Herpesvirus

## Abstract

*Genome signatures* are data vectors derived from the compositional statistics of DNA. The *self-organizing map* (SOM) is a neural network method for the conceptualisation of relationships within complex data, such as genome signatures. The various parameters of the SOM training phase are investigated for their effect on the accuracy of the resulting output map. It is concluded that larger SOMs, as well as taking longer to train, are less sensitive in phylogenetic classification of unknown DNA sequences. However, where a classification can be made, a larger SOM is more accurate. Increasing the number of iterations in the training phase of the SOM only slightly increases accuracy, without improving sensitivity. The optimal length of the DNA sequence *k*-mer from which the genome signature should be derived is 4 or 5, but shorter values are almost as effective. In general, these results indicate that small, rapidly trained SOMs are generally as good as larger, longer trained ones for the analysis of genome signatures. These results may also be more generally applicable to the use of SOMs for other complex data sets, such as microarray data.

## Introduction

Molecular evolutionary methodology revolves around the production of sequence alignments and trees. However, as evolutionary distance increases between two homologous molecules, their similarity may decay to the point where they are no longer alignable. Construction of a phylogenetic tree under such circumstances becomes impossible. One method that has been suggested for the study of distant evolutionary relationships is that of *genomic signatures* or *genome signatures*† (Karlin and Ladunga, 1994; Karlin and Burge, 1995; Karlin and Mrazek, 1996). At least one reviewer has come to the conclusion that it is the preferred method in cases where evolutionary distance, recombination, horizontal transmission or variable mutation rates may confound traditional alignment-based techniques (Brocchieri, 2001).

The first derivation of genome signatures predates the invention of DNA sequencing. Biochemical studies revealed that the frequencies of nearest-neighbour dinucleotide pairs in DNA were generally consistent within genomes, and often different between genomes. These characteristic nearest neighbour patterns were termed *general schemes* (Russell et al. 1976; Russell and Subak-Sharpe, 1977), and constitute, in modern terminology, a subset of genome signatures, those of length *k* = 2.

As long DNA sequences began to be isolated and computers entered the biological laboratory, it became a simple matter to produce nearest-neighbour frequency tables. Indeed, for any DNA sequence of length *N*, it is theoretically possible to derive frequency tables for all *k*-mers ranging from 1 to *N*, within that sequence. The frequency table at *k* = 1 corresponds to the raw nucleotide content on one strand. On the assumption that DNA is double stranded under most circumstances in most species, the complementary bases are also scored. This reduces the raw count of the four bases to a single value, between zero and one, representing the GC content of that DNA sequence. Correspondingly, at *k* = 2, the raw count of 16 dinucleotide frequencies, can be reduced to a vector containing 10 values if the count for each dimer on the top strand is added to the count for its complement on the other strand. There are 10 values, not 8, in this vector since GC, CG, AT and TA are self-complementary. This process is called *symmetrization* (Karlin and Ladunga, 1994). The symmetrized values in the vector are then usually corrected for the frequencies of their component monomers, as follows:

$$\rho_{XY}\;=\;{f_{XY}/f_{X}f}_{Y}$$

where *f*_XY_ is the symmetrized frequency of dinucleotide XY, and *f*_X_ and *f*_Y_ are the symmetrized frequencies of bases X and Y, respectively. The whole vector is referred to as the genome signature at *k* = 2 or, particularly in the extensive literature of the Karlin group, simply as ρ^*^ _XY._ For all values of *k*, the nomenclature GS-*k* is here adopted.

The vector thus becomes an array of the ratios of observed frequencies of *k*-mers to their expected frequencies given an underlying zero-order Markov chain model of a DNA sequence. Even though symmetrization will reduce the size of the vector for large values of *k*, it is apparent that it will still grow in size at the order of 4*^k^* for an alphabet of length 4. In practice, most investigators have confined themselves to the study of genome signatures of *k* = 2, in other words to ρ ^*^_XY_, symmetrized dinucleotide frequencies corresponding to general schemes, although in recent years the availability of faster computers has undoubtedly contributed to the increasing use of genome signatures up to *k* = 10 (Deschavanne et al. 1999; Edwards et al. 2002; Abe et al. 2003a; Sandberg et al. 2003; Campanaro et al. 2005; Dufraigne et al. 2005; Wang et al. 2005; Paz et al. 2006).

The length of DNA required to generate a genome signature has conventionally been taken to be around 50 kb, and for this value it has been observed that the Hamming or Euclidean distances between signatures derived from contigs *within* species are generally considerably smaller than the corresponding average values *between* species (Karlin and Ladunga, 1994; Karlin and Burge, 1995; Karlin et al. 1997; Abe et al. 2002; Teeling et al. 2004), even when the same-species contigs are on different chromosomes (Gentles and Karlin, 2001). However, recent work has established that genome signatures within species may be stable over lengths as short as 10 kb (Deschavanne et al. 1999; Karlin, 2001; Abe et al. 2002) or less (Sandberg et al. 2001; Jernigan and Baran, 2002; Abe et al. 2003a; Sandberg et al. 2003; McHardy et al. 2007). This has led to their practical application in the detection of *pathenogenicity islands* (pIs) in pathogenic bacteria. These are sequences originating in horizontal transmission from one bacterium to another, converting a previously innocuous strain into a pathogenic one. Their foreign origin is often reflected in a genome signature closer to their species of origin than their current host genome (Karlin, 1998; Karlin, 2001; Dufraigne et al. 2005).

Phylogenetic conclusions drawn from comparison of genome signatures have sometimes been controversial. For instance, Karlin et al. (1997) found that cyanobacteria do not form a coherent evolutionary group, and that *Methanococcus jannaschii* is closer to eukaryotes than to other proteobacteria, and Campbell et al. (1999) suggested that archaea do not form a coherent clade. Karlin (1998) posited a wide variety of further revisions of the prokaryotic phylogeny based on genome signature results, as well as a novel origin for mitochondria (Karlin et al. 1999). Edwards et al. (2002) used genome signatures as part of a revision of the phylogeny of birds. Nevertheless, few authors have felt confident enough to draw phylogenetic trees based on genome signature comparisons. Coenye and Vandamme (2004) have shown that dinucleotide content is only a reliable indicator of relatedness for closely related organisms. To visualize genome signature relationships between species, a variety of other representational schemes have been used including histograms (Karlin and Mrázek, 1997), partial ordering graphs (Karlin et al. 1997), chaos games (Deschavanne et al. 1999; Edwards et al. 2002; Wang et al. 2005), and self-organizing maps (Abe et al. 2003b).

This paper uses self-organizing maps (SOMs) as a tool to explore genome signature variability at phylogenetic levels from superkingdom down to genus. The SOM is a neural network method which spreads multi-dimensional data onto a two-dimensional surface (Kohonen, 1997). Its endpoint is therefore similar to multi-dimensional scaling or principal components analysis, and like these other techniques has been extensively used in biology, principally for the analysis of micro-array data but also to a lesser extent for sequence analysis (Arrigo et al. 1991; Giuliano et al. 1993; Andrade et al. 1997; Tamayo et al. 1999; Kanaya et al. 2001; Wang et al. 2001; Abe et al. 2002; Covell et al. 2003; Ressom et al. 2003; Xiao et al. 2003; Mahony et al. 2004; Oja et al. 2005; Abe et al. 2006; Samsonova et al. 2006). The resulting “flat” representation may be a strong aid to intuitive understanding of the structure of complex multidimensional datasets. The SOM is not a clustering technique *per se*, but the surface may be divided up into zones that are then treated as clusters. Alternatively, cluster boundaries on the surface may be defined more objectively using additional algorithms (Ultsch, 1993). The SOM is also not hierarchical (unlike UPGMA but like K-means clustering, two other commonly used techniques for the analysis of microarrays). This absence of hierarchy means that it is particularly suited to situations where the natural hierarchy of species relationships, reflecting evolutionary descent, may have been violated, e.g. by horizontal gene transfer.

In this paper, the main parameters of the SOM: its size and the number of iterations used in its construction, are investigated for their effects on its classificatory accuracy. These parameters must be chosen at the beginning of each run of SOM building, and there is little guidance in the SOM literature as to their optimal values. As well as the parameters of the SOM, the value of *k* used in the genome signature is similarly examined. High *k* genome signatures are extremely long vectors that may present considerable memory problems even on modern computers. Likewise, lengthy iterations in training the SOM, especially if it is a large one, may consume considerable time.

## Methods

### 1. Genome sequences

Complete genome sequences were downloaded from NCBI Taxonomy Browser (http://www.ncbi.nlm.nih.gov/Taxonomy/taxonomyhome.html/). A Perl script was written to divide complete genome sequences into consecutive strings of 10 or 100 kb, as required. Trailing ends, and genomes shorter than the required string length, were discarded. The resulting FASTA-formatted datasets were then processed to calculate their genome signatures.

**Figure 1.**
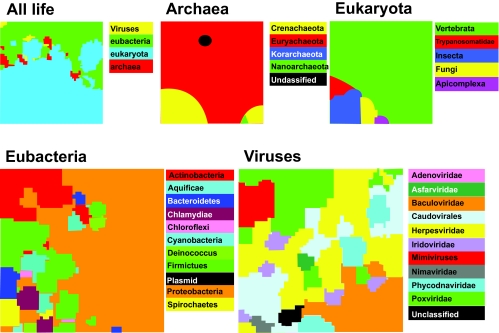
Dominance maps for GS-2 applied to a 50 ×50 SOM over 100 iterations. The eubacterial and viral SOMs are shown at a larger scale owing to their greater detail. Dominance areas are color coded.

**Figure 2.**
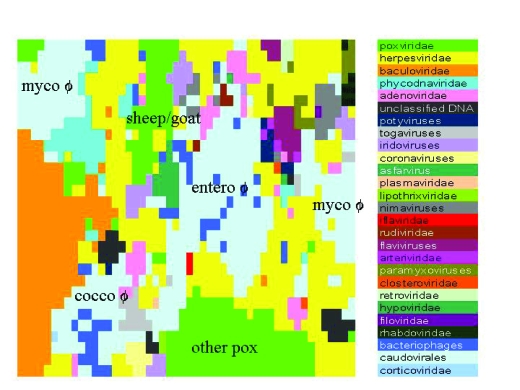
Dominance map for GS-2 of 10kb fragments of viruses applied to a 50 ×50 SOM over 1000 iterations. The category “Bacteriophages” refers to unclassified phages. Most phages are members of the family *Caudovirales*. The text added to the dominance map shows the general divisions of the *Poxviridae* and *Caudovirales* which form more than one well defined dominance area.

**Figure 3.**
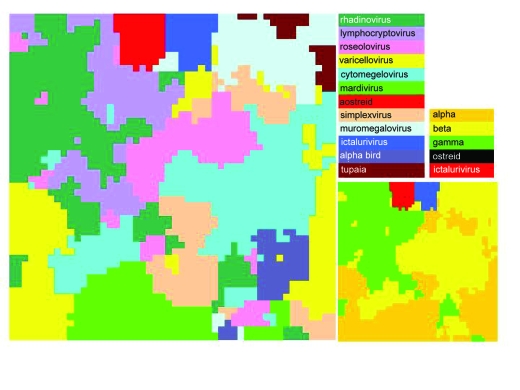
Dominance maps for GS-2 of 10kb fragments of herpesviruses applied to a 50 ×50 SOM over 500 iterations. The SOM is colored first according to genus membership and then according to family membership (reduced scale inset).

**Figure 4.**
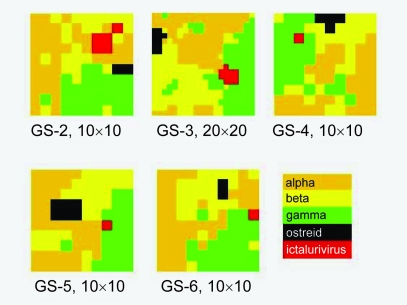
Dominance maps herpesvirus families, illustrating the effect of varying GS values using 10kb herpesvirus sequences, on a 10 ×10 SOM (except for GS-3 at 20 ×20) over 100 iterations.

**Figure 5.**
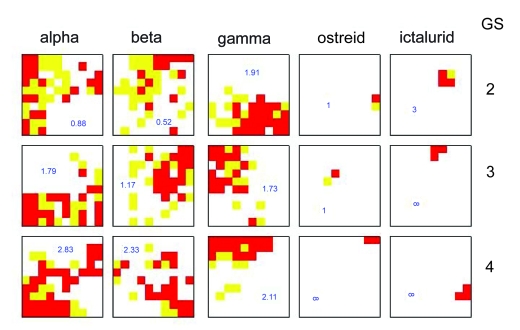
The density of herpesviral sequences, classed by family, on a 10 ×10 SOM after 100 iterations. >95% density: red; 5%–95% density: yellow; <5% density: white. The figure in each box is the ratio of sequences in red to yellow areas of the SOM.

**Figure 6.**
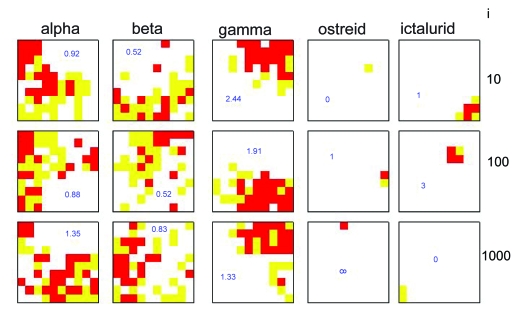
The density of herpesviral sequences, classed by family, on a 10 ×10 SOM of GS-2, run over a varying number of iterations, *i*. >95% density: red; 5%–95% density: yellow; <5% density: white. The figure in each box is the ratio of sequences in red to yellow areas of the SOM.

**Figure 7.**
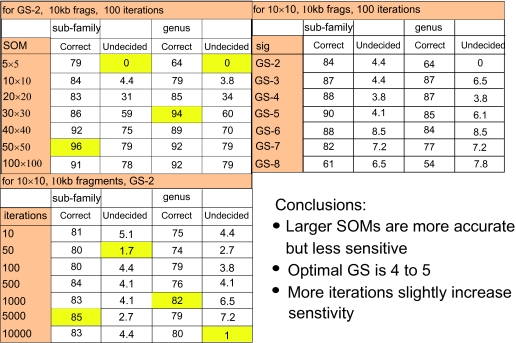
Jack-knifing experiments to determine effects of SOM size (top left), GS number (top right) and number of iterations (lower left). The “undecided” column indicates the percentage of sequences in the test set that could not be assigned to a sub-family or genus. The “correct” column indicates the percentage of assignable sequences that were correctly assigned. Optimal values are highlighted in yellow.

Table 1 lists the genomes used as the main data set for the paper, that of viruses of the family *Herpesviridae*. The analyses shown in Figures 3 to 7 use this set. A larger set of genomes with the widest possible phylogenetic range, including all three superkingdoms of cellular life as well as viruses, is given in Table 2. These are used for the “all-life” and superkingdom-level SOMs in Figure 1. Table 3 lists those viral genomes used for the SOM across a wide set of viral genomes, displayed in Figure 2.

### 2. Calculation of genome signatures

A Perl script was written to derive raw *k*-mer counts on FASTA-formatted databases of input sequences, using the SeqWords.pm module from BioPerl (http://www.bioperl.org/Pdoc-mirror/bioperl-live/Bio/Tools/SeqWords.html). The raw *k*-mer frequencies were then symmetrized, as follows:

$$f_{v}^{s}\;=\;f_{v}\;+\;f_{v-comp}$$

where f_ν_ and f_ν-comp_ are the raw frequencies of a *k*-mer ν and its complement ν-comp.

Symmetrization means that a sequence and its complement will generate the same answer. The symmetrized frequencies are then corrected for the 1-mer content. For instance for a 2-mer XY, where X and Y can each represent any nucleotide base {*A, C, T, G*}:

$$\rho_{XY}\;=\;f_{XY}^{s}/f_{X}^{s}f_{Y}^{s}$$

where f_sXY_ is the symmetrized frequency for dimer XY and f_sX_ and f_sY_ are the symmetrized frequencies of its component 1-mers. For a 3-mer XYZ, the correction would be for the 1-mers, X, Y and Z and so on.

The genome signature vector for length *k*, is thus composed of a series of ratios of observed to expected values of its component *k*-mers, where the expected values are determined by a zero-order Markov chain (Bernouilli series) model. Genome signatures are therefore not distorted by gross base compositional differences between genomes, which would otherwise be the dominant factor.

### 3. Self-organizing map

Self-organizing maps (SOMs) were run following Tamayo et al. (1999), using a Perl script. Input consisted of an array of the genome signatures generated as described above. The dimensions of the SOM and the number of iterations in training were variables entered by the user. Euclidean distances were used when comparing vectors.

Once the dimensions of the SOM were set, *x* columns by *y* rows, *weight vectors* initializing each of the *xy* cells of the SOM were selected at random from the entire set of genome signature data vectors. The SOM is thus initially simply filled with a random subset of the data. Training then commences, for nominated *t* iterations. At each iteration *m*, each data vector in turn was compared to each weight vector, and the closest weight vector for each data vector designated the *winning weight vector* of that data vector in that iteration. Each time a winning weight vector is identified, the winning weight vector, and the weight vectors of cells within a spatial range ℜ on the SOM, were then trained by the data vector as follows.

Each value *c* in the winning weight vector *w* is altered, so that its value at iteration, *m*, becomes at the next iteration *m*+1:

$$w_{m+1}^{c}\;=\;w_{m}^{c}\;+\;\tau_{m}\left(w_{m}^{c}\;-\;v^{c}\right)$$

where *w^c^m* – *v^c^* represents the difference between the winning weight vector and the data vector for each value *c* along the vectors. In other words, one simply aligns the data vector and the winning weight vector and subtracts them. Each value of the winning weight vector is then altered to bring it closer to the data vector by a factor of τ, the training effect, which is derived as follows:

$$\tau_{m}\;=\;\alpha_{m}/\gamma$$

τ changes at each iteration of the process, and is the ratio of two other values α and γ.

α is calculated for each iteration *m* as follows:

$$\alpha_{m}\;=\;1\;-\;\left(\frac{m\;-\;1}{t}\right)$$

where *m* is the number of the current iteration, and *t* the number of total iterations requested. There-fore, the number of iterations of the SOM, a parameter chosen at the start of the process, determines the gradient at which α will decrease as the iterations progress.

Whereas α is the same for all cells in the SOM and changes according to the iteration number only, γ is the Euclidean distance on the SOM from the weight vector being trained within range ℛ of the winning weight vector.

τ can therefore be seen to decrease as the SOM progresses, since α decreases, and also to decrease the further one goes away from the winning weight vector, since γ increases.

The range within which weight vectors are trained at each iteration is calculated:

$${ℜ}_{m}\;=\;\alpha_{m}S$$

where *S* is the length or breadth of the SOM, whichever is the smaller. The area of the SOM being trained therefore also shrinks as α decreases with increasing iterations.

Once each data vector has found its winning weight vector and trained it, also training the weight vectors within range ℜ of the winning weight vector, then one iteration is completed. New values of α,τ and ℜ are then calculated, and the second iteration can commence. It can be intuitively grasped that there is a great deal of “churn” in initial iterations of the SOM. When α is close to 1, data vectors will effectively change their winning weight vector to copies of themselves. Only at the limits of the trained area R will the effect be subtler. However, as the number of iterations mounts, α will decrease and each data vector will have a relatively weaker effect on its winning weight vector and even less on those weight vectors in its vicinity. Observation (data not shown) of distribution of a simple data set over a SOM through the iterative process shows that a relatively chaotic process dominates until approximately halfway through the nominated number of iterations, at which point structure rapidly builds in the SOM. The final 10% or so of iterations consist mostly of fine-tuning of the final weight vector values. Training SOMs can also be time consuming, especially for large data sets of high dimensionality vectors trained over large numbers of iterations. The longest run presented here (that in Fig. 2) took in excess of 3 weeks on a single 2.8 GHz Intel processor under a Linux operating system. One of the major motivations of this paper was to define ways to reduce SOM training time without losing accuracy or sensitivity.

After the final iteration, each data vector is again compared to each weight vector and assigned to the closest. This results in partition of each data vector to one cell in the SOM, thus spreading the multi-dimensional data across the two-dimensional surface of the SOM. Conversely, each final weight vector in the SOM is assigned to its closest data vector, the *centroid nearest neighbour* (cnn). If the data vectors belong to several categories, each cell in the SOM can be colored according to the origin of its cnn, which is then said to *dominate* that cell in the SOM. This allows the production of color-coded *dominance maps* indicating the general spread of the data vector set over the SOM. NCBI taxonomic categories were used throughout, except for herpesviruses where the International Committee on the Taxonomy of Viruses (ICTV) usage is followed (Davison 2002; Davison et al. 2005; Fauquet et al. 2005).

### 4. Availability of scripts

All Perl scripts, for processing genomes, calculating genome signatures, and running SOMs are available on request from the author ( d.gatherer@mrcvu.gla.ac.uk).

## Results

### 1. SOMs on large sequence datasets

The ability of SOMs to distinguish the origin of fragments of DNA based on their genome signatures, was initially tested using GS-2 (see Methods, section 2, above) measured over fragments of 100 kb. At the time of analysis there were 79 eukaryotic, 156 eubacterial, 30 archaeal and 122 viral genomes with more than 100 kb of sequence each (Table 2). The dimension of the SOM was 50 × 50 and 100 iterations were used. At the end of the iterations, dominance areas (see Methods, section 3, above), were used to color the SOM. For the entire data set, “all life” in Figure 1, the superkingdoms of archaea, eubacteria and eukaryota were chosen, along with the unranked category of viruses. Within each of the SOMs applied to the superkingdoms and the viruses, the next level down was used for coloring dominance maps. This is the phylum level in the archaea and eubacteria, and the family level in the viruses. In the eukaryota, the relative scarcity of completely sequenced genomes required a more *ad hoc* classification.

When all input sets are pooled, GS-2 produces a SOM in which eubacterial sequences cluster together (Fig. 1; “All life”, green). Archaeal sequences are split into several groups that are situated along the boundary between the eubacteria and the eukaryotes. Likewise, viral sequences are split into one group in the top left corner and other clusters along the eubacterial-eukaryotic border. It is evident that this “all life” SOM does not contribute to the issue of the phylogeny of the three superkingdoms, except to underline that archaea are not derivatives of either eukaryotes or eubacteria.

When the SOM is confined to archaeal sequences (Fig. 1; “Archaea”), those genomes designated “unclassified” by NCBI, are located well within the territory of the *Euryarchaeota*, strongly suggesting that they belong to this phylum. In general the archaeal inter-phylum boundaries are clear, although the *Crenarchaeaota* are split into two clusters. The predominance of *Euryarchaeota* in terms of area is a reflection of the larger number of complete genomes in that phylum.

Likewise, in the eukaryotes (Fig. 1; “Eukaryota”), the large size of the human genome contributes to a large area dominated by the *Vertebrata*. It should be remembered that the classification in the eukaryotes is *ad hoc* owing to the relatively small number of complete genomes. However, it is interesting that the boundaries between the dominance areas are as distinct as those in the archaea.

The situation is considerably more complicated within the eubacteria (Fig. 1; “Eubacteria”), being the superkingdom with the greatest number of completely sequenced genomes. Some eubacterial phyla are rather fragmented in their dominance areas. For instance, the phylum *Firmicutes* occupies several partly adjacent areas. The phylum *Deinococcus* has two small and rather distant dominance areas, and the *Bacteroidetes* and *Spirochaetes* both have small outlying fragments. The *Proteobacteria* dominate the right side of the SOM and penetrate between the various groups on the left side. The overall impression is of less clear-cut differences in GS-2 between phyla in eubacteria than in eukaryotes or archaea.

A similar situation is observed in the SOM on viral sequences (Fig. 1; “Viruses”). A few viral families, such as the *Baculoviridae*, the family *Mimivirus* and the *Nimaviridae* do manage coherent dominance areas, but all others are extensively mixed. The *Baculoviridae* are the only family of any size than maintain a distinctive dominance area.

This basic illustration of the SOM in action demonstrates that for a single parameter set, namely 50 × 50 SOM and 100 iterations, different phylogenetic groups exhibit variable degrees of partition across the SOM.

### 2. Increased resolution SOM on viruses

To increase the resolution of the SOM against viral sequences, GS-2 was reapplied to viral sequences only using 10 kb fragments. This enables a larger number of viral genomes to be analysed, up from 122 to 579, as genomes of 10 kb or more can be included (Table 3). The number of iterations was increased to 1000. The resulting dominance map is shown in Figure 2.

When viral sequences alone are considered at higher resolution, the SOM becomes very complex. The family level classification is maintained for the dominance map but there are now more families, since viruses as small as 10 kb are eligible. Perhaps the most salient feature is that *Poxviridae* are divisible into sheep/goat pox viruses and others (Fig. 2: “sheep/goat” and “other pox”). Additionally phages, within the family *Caudovirales*, tend to be differentially located on the SOM in four major areas, one of which, mycophages, accounts for two of these areas (Fig. 2: “myco-ϕ”, “entero-ϕ” and “cocco-ϕ”). Again the *Baculoviridae* form a noticeably large and coherent cluster. *Herpesviridae*, by contrast, are spread across the entire map.

*Herpesviridae* (Table 1) are next considered alone under the same conditions as in Figure 2. Dominance maps for this narrower selection are shown in Figure 3.

Figure 3 shows that when family-level taxonomy is considered within herpesviruses, GS-2 distinguishes the ostreid herpesviruses and the ictaluriviruses as two fairly homogenous blocs distinct from the *Alloherpesviridae* (Davison, 2002), comprising the alpha, beta and gamma families. At the genus-level, *Muromegalovirus* alone forms a nearly contiguous bloc although *Mardivirus* nearly does so. The remaining genera, like the families, are considerably mixed across the SOM. Like the wide spread of herpesvirus signatures across the viral SOM, this is a reflection of the degree of sequence heterogeneity with the *Herpesviridae*.

The three figures presented above demonstrate that the SOM is an intriguing tool for the conceptualisation of relationships between genome signatures. However, the evident complexity of some of the topographical arrangements raises serious questions concerning its utility as a diagnostic tool for phylogeny.

Therefore, some experiments are described which address this issue in a quantitative way.

### 3. Effect of length of *k*-mer used to generate genome signature

In order to investigate if genome signatures of longer *k* give better resolution than *k* = 2, 10 kb herpesvirus sequences were processed into genome signature of GS-2 to GS-6 and the SOM was trained for 100 iterations (Fig. 4). On first inspection, it does not appear that a higher genome signature provides any better resolution than a lower one. The GS-3 SOM was also run on a 20 × 20 map, but again this produces no major change to the overall pattern. In all cases, ostreid herpesvirus and ictalurivirus have coherent dominance areas on the SOM. At GS-5, alpha herpes-viruses also have a coherent dominance area, but this disappears again at GS-6. In order to further investigate this apparent lack of improvement at higher values of *k*, the density of sequences of each family was plotted onto the SOM (Fig. 5). Instead of the dominance map approach, in which each cell is colored according to the affiliation of its cnn (Fig. 1–4 are all of this type), cells in which more than 95% of allocated sequences are of a single type are colored red, and those with fewer than 5% of that type are white. Cells between these two extremes are colored yellow. A ratio is then produced of red-to-yellow in each SOM. A perfectly partitioned SOM will therefore have a ratio of infinity, indicating no mixed cells, or more accurately no cells with greater than 5% mixture of the “wrong” family.

Figure 5 demonstrates that family level taxonomy is better determined at higher GS in all five families of herpesviruses. The ratio of high alpha-density (>95%, red) to medium alpha-density (5% to 95%, yellow) increases from 0.88 to 2.83 as the GS increases from 2 to 4. The corresponding increases for the beta and gamma families are from 0.52 to 2.33 and from 1.91 to 2.11 respectively. For the ostreid herpesviral sequences, perfect partition is reached at GS-4 and for the ictalurid viruses at GS-3. This is probably a reflection of the presence of a single virus in each of these categories with a correspondingly lower number of sequences analysed.

### 4. Effect of length of training phase of SOM

It is therefore apparent that genome signature of longer values of *k* produce some improvement in the accuracy of the final partition on the SOM. However, longer *k* results in longer data vectors, increasing at order 4*^k^* and therefore much slower training of the SOM. One way to speed training of the SOM is simply to reduce the number of training cycles. The effect of the number of iterations on density of each family is displayed in Figure 6.

Figure 6 shows that increasing the number of iterations has a mixed effect on the density of family sequences. The alpha herpesviral sequences increase in density from 0.92 to 1.35 as the number of iterations increases from 10 to 1000, and the beta herpesviruses from 0.52 to 0.83. The ostreid herpesviral sequences are also perfectly clustered at 100 iterations. However, the gamma and ictalurid sequences are more poorly partitioned at higher numbers of iterations.

### 5. Jack-knifing analysis

Figures 1–6 provide a largely qualitative impression of the effectiveness of SOMs in correctly assigning the origins of DNA sequences based on their genome signature. To provide a further more quantitative assessment of the parameters of the process, a jack-knifing analysis was carried out. All herpesviral sequences were divided randomly into two groups. Genome signatures and SOMs were constructed as appropriate using one half. Then the remaining half was applied to the SOM to predict their origin at the family and genus level. To make a prediction concerning the origin of a data vector, the Euclidean distances between that vector and all of the weight vectors of the preconstructed SOM, are calculated. The origin of the nearest weight vector is taken to be the classification of the data vector being tested. Where a data vector falls into a cell on the SOM containing none of the original data vectors used to construct the SOM, its origin is deemed to be “undecided” (Fig. 7).

When SOM size is varied for GS-2 at 100 iterations (Fig. 7, top left table), SOMs of greater than 10 × 10 introduce considerably uncertainty into the assignment. However, for those sequences that can be assigned, 95% accuracy at the subfamily level is achieved in a 50 × 50 SOM. Likewise, a 30 × 30 SOM gives 94% accuracy at the genus level. When SOM size is held at 10 × 10 and the signature length at GS-2 and the number of iterations is varied (Fig. 7, lower left table), there is little effect on the sensitivity. At the subfamily level, there are never more than 4.4% of sequences that cannot be assigned, and never more than 7.2% at the genus level. Where sequences can be assigned, optimal accuracy is achieved at 1000 or 5000 iterations, but the variation in accuracy is low. Increasing the iterations from 10 to 5000 only gives a 4% increase in accuracy of assignment at the sub-family level. When 100 iterations are used and the SOM size is held at 10 × 10 (Fig. 7, top right table), GS-4 or GS-5 appear to be optimal.

## Discussion

Genome signatures provide a summary of the *k*-mer content of a genome, corrected for compositional bias. Various studies in a wide range of species have revealed that genome signatures are generally constant within genomes and similar in related genomes (Karlin and Ladunga, 1994; Karlin et al. 1998; Gentles and Karlin, 2001). The extent to which this is a phenomenon of neutral drift or one of active conservation is unknown. It is intuitively obvious that two identical genomes will have identical genome signatures, and that as they diverge the genome signatures will also diverge. Indeed this is the basis of a least one bioinformatical tool that assesses sequence relatedness (Li et al. 2001; Li et al. 2002). However, various suggestions have been made for conservative selection pressures which would act to maintain genome signature similarity in related organisms, including dinucleotide stacking energies, curvature, methylation, superhelicity, context-dependent mutation biases and effects deriving from related replication machinery (Karlin and Burge, 1995; Blaisdell et al. 1996). If these factors are similar within a clade, they might act as a brake on genome signature divergence. The conservation of genome signatures within genomes (which is what originally gave rise to the term “signature” in this context) would tend to suggest that signatures do not drift neutrally, at least *within* genomes.

Figure 1 demonstrates that at the phylum level within the three superkingdoms of cellular life, satisfactory partition of GS-2 can be obtained by the SOM. However, this is less true for eubacteria than it is for eukaryotes and archaea. At the family level in viruses the picture is considerably more confused, with only the *Baculoviridae* demonstrating anything like territorial coherence on the SOM at GS-2 (Fig. 1 and 2). This may well be a reflection of speed of substitution in viral genomes. However at the species level, the same coherence within genomes as found in cellular organisms may well be the norm. For instance, when the ostreid and ictalurid herpes-virus families are included in a SOM with the *Alloherpesviridae*, these two families, both represented by a single viral genome, have strongly discrete areas on the SOM (Fig. 3 and 4).

This does not mean that genome signatures are not diagnostic tools for phylogenetic assignment at the family and sub-family level in herpesviruses, merely that the results should be interpreted with caution. The use of higher values of *k* appears to have a marginal effect on improving the discrete distribution of family-level herpesviral signatures on the SOM (Fig. 5) but jack-knifing indicates that this does not improve above *k* = 5 (Fig. 7). The effects of larger dimension SOMs and increased iterations are ambiguous at best. Optimal values appear to be around GS-4 or GS-5 with 500 to 1000 iterations of the SOM. The size of the SOM might be varied, with an initial run at high dimension (e.g. 50 × 50) followed by a lower dimension run (e.g. 10 × 10) for sequences unassigned by the first run (Fig. 7).

The use of genome signatures in the identification of pathogenicity islands is by now well established (Karlin, 1998; Karlin, 2001; Dufraigne et al. 2005). They are valuable in this context in that they indicate regions within genomes that have characteristics different to the rest of the genome. However, it is apparent from the present work that it is difficult on the basis of genome signatures to accurately identify the origin of the exogenous DNA. A BLAST search is more likely to generate informative hits in this context. Nevertheless for sequences that cannot be precisely identified on the basis of alignment-based methods such as BLAST, genome signatures with SOMs holds out the prospect of identification of origin to a reasonable level.

The optimization of SOM parameters reported here may also extend to other applications of SOMs. Of particular interest in bioinformatics is their use for the analysis of microarray data. The experimental design would be the same, with a standard microarray data set (e.g. the breast cancer data provided by Reid et al. 2005) substituting for the genome signature arrays. Dominance mapping would be done by clinical outcome, and jack-knife analysis could test the accuracy and sensitivity of assignment of that outcome.

## Figures and Tables

**Table 1.** Herpesvirus genome sequences used for the analyses shown in Figures 3 to 7. The nomenclature follows the International Committee on Taxonomy of Viruses (Fauquet et al. 2005).

**Name**	**Accession**	**Sub-family**	**Genus**
Psittacid herpesvirus 1	NC_005264	Alpha	Iltovirus
Gallid herpesvirus 2	NC_002229	Alpha	Mardivirus
Gallid herpesvirus 3	NC_002577	Alpha	Mardivirus
Meleagrid herpesvirus 1	NC_002641	Alpha	Mardivirus
Cercopithecine herpesvirus 1	NC_004812	Alpha	Simplexvirus
Human herpesvirus 1	NC_001806	Alpha	Simplexvirus
Human herpesvirus 2	NC_001798	Alpha	Simplexvirus
Bovine herpesvirus 1	NC_001847	Alpha	Varicellovirus
Bovine herpesvirus 5	NC_005261	Alpha	Varicellovirus
Cercopithecine herpesvirus 7	NC_002686	Alpha	Varicellovirus
Equid herpesvirus 1	NC_001491	Alpha	Varicellovirus
Equid herpesvirus 4	NC_001844	Alpha	Varicellovirus
Human herpesvirus 3	NC_001348	Alpha	Varicellovirus
Suid herpesvirus 1	NC_006151	Alpha	Varicellovirus
Cercopithecine herpesvirus 8	NC_006150	Beta	Cytomegalovirus
Chimpanzee cytomegalovirus	NC_003521	Beta	Cytomegalovirus
Human herpesvirus 5 (AD169)	NC_001347	Beta	Cytomegalovirus
Human herpesvirus 5 (Merlin)	NC_006273	Beta	Cytomegalovirus
Murid herpesvirus 1	NC_004065	Beta	Muromegalovirus
Murid herpesvirus 2	NC_002512	Beta	Muromegalovirus
Human herpesvirus 6	NC_001664	Beta	Roseolovirus
Human herpesvirus 6B	NC_000898	Beta	Roseolovirus
Human herpesvirus 7	NC_001716	Beta	Roseolovirus
Tupaia herpesvirus	NC_002794	Beta	Tupaiavirus
Callitrichine herpesvirus 3	NC_004367	Gamma	Lymphocryptovirus
Cercopithecine herpesvirus 15	NC_006146	Gamma	Lymphocryptovirus
Human herpesvirus 4	NC_001345	Gamma	Lymphocryptovirus
Alcelaphine herpesvirus 1	NC_002531	Gamma	Rhadinovirus
Ateline herpesvirus 3	NC_001987	Gamma	Rhadinovirus
Bovine herpesvirus 4	NC_002665	Gamma	Rhadinovirus
Cercopithecine herpesvirus 17	NC_003401	Gamma	Rhadinovirus
Equid herpesvirus 2	NC_001650	Gamma	Rhadinovirus
Human herpesvirus 8	NC_003409	Gamma	Rhadinovirus
Murid herpesvirus 4	NC_001826	Gamma	Rhadinovirus
Saimiriine herpesvirus 2	NC_001350	Gamma	Rhadinovirus
Ictalurid herpesvirus 1	NC_001493	unassigned	Ictalurivirus
Ostreid herpesvirus 1	NC_005881	unassigned	unassigned

**Table 2.** Genomes used for the analysis shown in Figure 1. In total there are 79 eukaryotic, 156 eubacterial, 30 archaeal and 122 viral genomes with more than 100kb of sequence.

**Name**	**Superkingdom**	**Accession**
Aeropyrum pernix K1	archaea	NC_000854
Archaeoglobus fulgidus DSM 4304	archaea	NC_000917
cf. Archaea SAR-1	archaea	NS_000019
Ferroplasma acidarmanus Type I	archaea	NS_000030
Ferroplasma sp. Type II	archaea	NS_000029
Haloarcula marismortui ATCC43049 chromosome I	archaea	NC_006396
Haloarcula marismortui ATCC43049 chromosome II	archaea	NC_006397
Halobacterium sp. NRC-1	archaea	NC_002607
Halobacterium sp. NRC-1 plasmid pNRC100	archaea	NC_001869
Methanocaldococcus jannaschii DSM2661	archaea	NC_000909
Methanococcus maripaludis S2	archaea	NC_005791
Methanopyrus kandleri AV19	archaea	NC_003551
Methanosarcina acetivorans C2A	archaea	NC_003552
Methanosarcina barkeri str. fusaro chromosome 1	archaea	NC_007355
Methanosarcina mazei Go1	archaea	NC_003901
Methanothermobacter thermautotrophicus str. DeltaH	archaea	NC_000916
Nanoarchaeum equitans Kin4-M	archaea	NC_005213
Natronomonas pharaonis DSM2160	archaea	NC_007426
Picrophilus torridus DSM9790	archaea	NC_005877
Pyrobaculum aerophilum str. IM2	archaea	NC_003364
Pyrococcus abyssi GE5	archaea	NC_000868
Pyrococcus furiosus DSM3638	archaea	NC_003413
Pyrococcus horikoshii OT3	archaea	NC_000961
Sulfolobus acidocaldarius DSM639	archaea	NC_007181
Sulfolobus solfataricus P2	archaea	NC_002754
Sulfolobus tokodaii str. 7	archaea	NC_003106
Thermococcus kodakaraensis KOD1	archaea	NC_006624
Thermoplasma acidophilum DSM1728	archaea	NC_002578
Thermoplasma volcanium GSS1	archaea	NC_002689
Thermoplasmatales archaeon Gpl	archaea	NS_000033
Agrobacterium tumefaciens str. C58	eubacteria	NC_003062
Anabaena variabilis ATCC 29413	eubacteria	NC_007413
Aquifex aeolicus VF5	eubacteria	NC_000918
Azoarcus sp. EbN1	eubacteria	NC_006513
Bacillus cereus ATCC 10987	eubacteria	NC_003909
Bacillus cereus E33L	eubacteria	NC_006274
Bacillus subtilis sub sp. subtilis str. 168	eubacteria	NC_000964
Bacteroides fragilis NCTC9343	eubacteria	NC_003228
Bacteroides fragilis YCH46	eubacteria	NC_006347
Bartonella henselae str. Houston-1	eubacteria	NC_005956
Bartonella quintana str. Toulouse	eubacteria	NC_005955
BBUR Borrelia burgdorferi B31	eubacteria	NC_001318
Bifidobacterium longum NCC2705	eubacteria	NC_004307
Bordetella parapertussis 12822	eubacteria	NC_002928
Bordetella pertussis TohamaI	eubacteria	NC_002929
Bradyrhizobium japonicum USDA110	eubacteria	NC_004463
Brucella abortus biovar 1 str. 9-941 chromosome I	eubacteria	NC_006932
Brucella abortus biovar 1 str. 9-941 chromosome II	eubacteria	NC_006933
Brucella suis 1330 chromosome I	eubacteria	NC_004310
Buchnera aphidicola str. APS (Acyrthosiphonpisum)	eubacteria	NC_002528
Buchnera aphidicola str. Sg (Schizaphisgraminum)	eubacteria	NC_004061
Burkholderia mallei ATCC23344 chromosome 1	eubacteria	NC_006348
Burkholderia mallei ATCC23344 chromosome 2	eubacteria	NC_006349
Burkholderia pseudomallei 1710b chromosome I	eubacteria	NC_007434
Burkholderia pseudomallei 1710b chromosome II	eubacteria	NC_007435
Burkholderia pseudomallei K96243 chromosome 1	eubacteria	NC_006350
Burkholderia sp. 383 chromosome 1	eubacteria	NC_007510
Burkholderia sp. 383 chromosome 2	eubacteria	NC_007511
Burkholderia sp. 383 chromosome 3	eubacteria	NC_007509
Candidatus Blochmannia pennsylvanicus str. BPEN	eubacteria	NC_007292
Candidatus Pelagibacter ubique HTCC1062	eubacteria	NC_007205
Carboxydothermus hydrogenoformans Z-2901	eubacteria	NC_007503
Caulobacter crescentus CB15	eubacteria	NC_002696
Chlamydia trachomatis A/HAR-13	eubacteria	NC_007429
Chlamydia trachomatis D/UW-3/CX	eubacteria	NC_000117
Chlamydophila caviae GPIC	eubacteria	NC_003361
Chlamydophila pneumoniae AR39	eubacteria	NC_002179
Chlamydophila pneumoniae CWL029	eubacteria	NC_000922
Chlamydophila pneumoniae J138	eubacteria	NC_002491
Chlorobium chlorochromatii CaD	eubacteria	NC_007514
Clostridium acetobutylicum ATCC824	eubacteria	NC_003030
Clostridium tetani E88	eubacteria	NC_004557
Colwellia psychrerythraea 34H	eubacteria	NC_003910
Corynebacterium glutamicum ATCC13032	eubacteria	NC_003450
Corynebacterium jeikeium K411	eubacteria	NC_007164
Coxiella burnetii RSA493	eubacteria	NC_002971
Dechloromonas aromatica RCB	eubacteria	NC_007298
Dehalococcoides sp. CBDB1	eubacteria	NC_007356
Deinococcus radiodurans R1 chromosome 1	eubacteria	NC_001263
Deinococcus radiodurans R1 chromosome 2	eubacteria	NC_001264
Desulfovibrio vulgaris sub sp. vulgaris str. Hildenborough	eubacteria	NC_002937
Desulfovibriode sulfuricans G20	eubacteria	NC_007519
Ehrlichia canis str. Jake	eubacteria	NC_007354
Erwinia carotovora sub sp. atrosepticaSCRI1043	eubacteria	NC_004547
Escherichia coli CFT073	eubacteria	NC_004431
Escherichia coli K12	eubacteria	NC_000913
Escherichia coli O157:H7EDL933	eubacteria	NC_002655
Francisella tularensis sub sp. tularensis Schu4	eubacteria	NC_006570
Geobacter metallireducens GS-15	eubacteria	NC_007517
Haemophilus ducreyi 35000HP	eubacteria	NC_002940
Haemophilus influenzae 86-028NP	eubacteria	NC_007146
Haemophilus influenzae RdKW20	eubacteria	NC_000907
Helicobacter pylori 26695	eubacteria	NC_000915
Helicobacter pylori J99	eubacteria	NC_000921
Leifsoniaxyli sub sp. xyli str. CTCB07	eubacteria	NC_006087
Leptospira interrogans serovar Copenhageni chromosome I	eubacteria	NC_005823
Leptospira interrogans serovar Copenhageni chromosome II	eubacteria	NC_005824
Leptospira interrogans serovar Lai str. 56601 chromosome I	eubacteria	NC_004342
Mannheimia succiniciproducens MBEL55E	eubacteria	NC_006300
Mesoplasma florum L1	eubacteria	NC_006055
Mesorhizobium loti MAFF303099	eubacteria	NC_002678
Methylococcus capsulatus str. Bath	eubacteria	NC_002977
Mycobacterium avium sub sp. paratuberculosis K-10	eubacteria	NC_002944
Mycobacterium bovis AF2122/97	eubacteria	NC_002945
Mycobacterium leprae TN	eubacteria	NC_002677
Mycobacterium tuberculosis H37Rv	eubacteria	NC_000962
Mycoplasma genitalium G-37	eubacteria	NC_000908
Mycoplasma hyopneumoniae 7448	eubacteria	NC_007332
Mycoplasma hyopneumoniae J	eubacteria	NC_007295
Mycoplasma synoviae 53	eubacteria	NC_007294
Neisseria gonorrhoeae FA1090	eubacteria	NC_002946
Neisseria meningitidis MC58	eubacteria	NC_003112
Neisseria meningitidis Z2491	eubacteria	NC_003116
Nitrobacter winogradskyi Nb-255	eubacteria	NC_007406
Nitrosococcus oceani ATCC 19707	eubacteria	NC_007484
Nitrosomonas europaea ATCC 19718	eubacteria	NC_004757
Nocardia farcinicaI FM10152	eubacteria	NC_006361
Oceanobacillus iheyensis HTE831	eubacteria	NC_004193
Parachlamydia sp. UWE25	eubacteria	NC_005861
Pasteurella multocida sub sp. multocida str. Pm70	eubacteria	NC_002663
Pelobacter carbinolicus DSM2380	eubacteria	NC_007498
Pelodictyon luteolum DSM273	eubacteria	NC_007512
Photobacterium profundum SS9 chromosome 1	eubacteria	NC_006370
Photobacterium profundum SS9 chromosome 2	eubacteria	NC_006371
Photorhabdus luminescens sub sp. laumondii TTO1	eubacteria	NC_005126
Prochlorococcus marinus str. NATL2A	eubacteria	NC_007335
Prochlorococcus marinus sub sp. pastoris str. CCMP1986	eubacteria	NC_005072
Propionibacterium acnes KPA171202	eubacteria	NC_006085
Pseudoalteromonas haloplanktis TAC125 chromosome I	eubacteria	NC_007481
Pseudoalteromonas haloplanktis TAC125 chromosome II	eubacteria	NC_007482
Psuedomonas fluorescens Pf-5	eubacteria	NC_004129
Psuedomonas fluorescens PfO-1	eubacteria	NC_007492
Psuedomonas putida KT2440	eubacteria	NC_002947
Psuedomonas syringae pv. phaseolicola 1448A	eubacteria	NC_005773
Psuedomonas syringae pv. syringae B728a	eubacteria	NC_007005
Psuedomonas syringae pv. tomato str. DC3000	eubacteria	NC_004578
Psychrobacter arcticus 273-4	eubacteria	NC_007204
Ralstonia eutropha JMP134 chromosome 1	eubacteria	NC_007347
Ralstonia eutropha JMP134 chromosome 2	eubacteria	NC_007348
Ralstonia solanacearum GMI1000	eubacteria	NC_003295
Rhodobacter sphaeroides 2.1 chromosome 1	eubacteria	NC_007493
Rhodobacter sphaeroides 2.1 chromosome 2	eubacteria	NC_007494
Rickettsia conorii str. Malish 7	eubacteria	NC_003103
Rickettsia felis URRWXCal2	eubacteria	NC_007109
Rickettsia prowazekii str. MadridE	eubacteria	NC_000963
Rickettsia typhi str. Wilmington	eubacteria	NC_006142
Salmonella enterica serovar Choleraesuis str. SC-B67	eubacteria	NC_006905
Salmonella enterica serovar Typhi str. CT18	eubacteria	NC_003198
Shewanella oneidensis MR-1	eubacteria	NC_004347
Shigella flexneri 2a str. 2457T	eubacteria	NC_004741
Shigella flexneri 2a str. 301	eubacteria	NC_004337
Shigella sonnei Ss046	eubacteria	NC_007384
Sinorhizobium meliloti 1021	eubacteria	NC_003047
Staphylococcus aureus sub sp. Aureus Mu50	eubacteria	NC_002758
Staphylococcus haemolyticus JCSC143	eubacteria	NC_007168
Staphylococcus saprophyticus sub sp. saprophyticus	eubacteria	NC_007350
Streptococcus agalactiae A909	eubacteria	NC_007432
Streptococcus pyogenes MGAS10394	eubacteria	NC_006086
Streptococcus pyogenes MGAS315	eubacteria	NC_004070
Streptococcus pyogenes MGAS500	eubacteria	NC_007297
Streptococcus pyogenes MGAS6180	eubacteria	NC_007296
Streptococcus pyogenes SSI-1	eubacteria	NC_004606
Streptococcus thermophilus CNRZ1066	eubacteria	NC_006449
Streptococcus thermophilus LMG18311	eubacteria	NC_006448
Streptomyces avermitilis MA-4680	eubacteria	NC_003155
Streptomyces coelicolor A3(2)	eubacteria	NC_003888
Synechococcus sp. CC9605	eubacteria	NC_007516
Synechococcus sp. CC9902	eubacteria	NC_007513
Thermobifida fusca YX	eubacteria	NC_007333
Thermus thermophilus HB8	eubacteria	NC_006461
Thiobacillus denitrificans ATCC2525	eubacteria	NC_007404
Thiomicrospira crunogena XCL-2	eubacteria	NC_007520
Tropheryma whipplei str. Twist	eubacteria	NC_004572
Vibrio cholerae O1 biovar eltor str. N16961 chromosome I	eubacteria	NC_002505
Vibrio vulnificus CMCP6 chromosome I	eubacteria	NC_004459
Vibrio vulnificus CMCP6 chromosome II	eubacteria	NC_004460
Wolbachia endosymbiont strain TRS of Brugiamalayi	eubacteria	NC_006833
Wolinella succinogenes DSM1740	eubacteria	NC_005090
Xanthomonas axonopodis pv. citri str. 306	eubacteria	NC_003919
Xanthomonas campestris pv. campestris str. 8004	eubacteria	NC_007086
Xanthomonas campestris pv. campestris str. ATCC33913	eubacteria	NC_003902
Xanthomonas campestris pv. vesicatoria str. 85-10	eubacteria	NC_007508
Xanthomonas oryzae pv. oryzae KACC10331	eubacteria	NC_006834
Xylella fastidiosa 9a5c	eubacteria	NC_002488
Xylella fastidiosa Temecula 1	eubacteria	NC_004556
Yersinia pseudotuberculosis IP32953	eubacteria	NC_006155
Bos taurus genome 12	eukaryote	NC_007310
Bos taurus genome 13	eukaryote	NC_007311
Bos taurus genome 14	eukaryote	NC_007312
Bos taurus genome 15	eukaryote	NC_007313
Bos taurus genome 16	eukaryote	NC_007314
Bos taurus genome 17	eukaryote	NC_007315
Bos taurus genome 18	eukaryote	NC_007316
Bos taurus genome 19	eukaryote	NC_007317
Bos taurus genome 20	eukaryote	NC_007318
Bos taurus genome 21	eukaryote	NC_007319
Bos taurus genome 22	eukaryote	NC_007320
Bos taurus genome 23	eukaryote	NC_007324
Bos taurus genome 24	eukaryote	NC_007325
Bos taurus genome 25	eukaryote	NC_007326
Bos taurus genome 26	eukaryote	NC_007327
Bos taurus genome 27	eukaryote	NC_007328
Bos taurus genome 28	eukaryote	NC_007329
Bos taurus genome 29	eukaryote	NC_007330
Bos taurus genome X	eukaryote	NC_007331
Candida albicans genomic DNA, genome 7	eukaryote	NC_007436
Cryptococcus neoformans genome 1	eukaryote	NC_006670
Cryptococcus neoformans genome 10	eukaryote	NC_006679
Cryptococcus neoformans genome 11	eukaryote	NC_006680
Cryptococcus neoformans genome 12	eukaryote	NC_006681
Cryptococcus neoformans genome 13	eukaryote	NC_006682
Cryptococcus neoformans genome 14	eukaryote	NC_006683
Cryptococcus neoformans genome 2	eukaryote	NC_006684
Cryptococcus neoformans genome 3	eukaryote	NC_006685
Cryptococcus neoformans genome 4	eukaryote	NC_006686
Cryptococcus neoformans genome 5	eukaryote	NC_006687
Cryptococcus neoformans genome 6	eukaryote	NC_006691
Cryptococcus neoformans genome 7	eukaryote	NC_006692
Cryptococcus neoformans genome 8	eukaryote	NC_006693
Cryptococcus neoformans genome 9	eukaryote	NC_006694
Cryptosporidium parvum genome 1	eukaryote	NC_006980
Cryptosporidium parvum genome 2	eukaryote	NC_006981
Cryptosporidium parvum genome 3	eukaryote	NC_006982
Cryptosporidium parvum genome 4	eukaryote	NC_006983
Cryptosporidium parvum genome 5	eukaryote	NC_006984
Cryptosporidium parvum genome 6	eukaryote	NC_006985
Cryptosporidium parvum genome 7	eukaryote	NC_006986
Cryptosporidium parvum genome 8	eukaryote	NC_006987
Drosophila melanogaster genome 2L	eukaryote	NT_033779
Drosophila melanogaster genome 2R	eukaryote	NT_033778
Drosophila melanogaster genome 3L	eukaryote	NT_037436
Drosophila melanogaster genome 3R	eukaryote	NT_033777
Drosophila melanogaster genome 4	eukaryote	NC_004353
Drosophila melanogaster genome X	eukaryote	NC_004354
Leishmania major strain Friedlin genome 27	eukaryote	NC_007268
Leishmania major strain Friedlin genome 29	eukaryote	NC_007270
Leishmania major strain Friedlin genome 4	eukaryote	NC_007245
Saccharomyces cerevisiae genome I	eukaryote	NC_001133
Saccharomyces cerevisiae genome II	eukaryote	NC_001134
Saccharomyces cerevisiae genome III	eukaryote	NC_001135
Saccharomyces cerevisiae genome IV	eukaryote	NC_001136
Saccharomyces cerevisiae genome IX	eukaryote	NC_001141
Saccharomyces cerevisiae genome V	eukaryote	NC_001137
Saccharomyces cerevisiae genome VI	eukaryote	NC_001138
Saccharomyces cerevisiae genome VII	eukaryote	NC_001139
Saccharomyces cerevisiae genome VIII	eukaryote	NC_001140
Saccharomyces cerevisiae genome X	eukaryote	NC_001142
Saccharomyces cerevisiae genome XI	eukaryote	NC_001143
Saccharomyces cerevisiae genome XII	eukaryote	NC_001144
Saccharomyces cerevisiae genome XIII	eukaryote	NC_001145
Saccharomyces cerevisiae genome XIV	eukaryote	NC_001146
Saccharomyces cerevisiae genome XV	eukaryote	NC_001147
Saccharomyces cerevisiae genome XVI	eukaryote	NC_001148
Trypanosoma brucei TREU927 genome 1	eukaryote	NC_007334
Trypanosoma brucei TREU927 genome 10	eukaryote	NC_007283
Trypanosoma brucei TREU927 genome 11 scaffold 1	eukaryote	NT_165288
Trypanosoma brucei TREU927 genome 2	eukaryote	NC_005063
Trypanosoma brucei TREU927 genome 3	eukaryote	NC_007276
Trypanosoma brucei TREU927 genome 4	eukaryote	NC_007277
Trypanosoma brucei TREU927 genome 5	eukaryote	NC_007278
Trypanosoma brucei TREU927 genome 6	eukaryote	NC_007279
Trypanosoma brucei TREU927 genome 7	eukaryote	NC_007280
Trypanosoma brucei TREU927 genome 8	eukaryote	NC_007281
Trypanosoma brucei TREU927 genome 9	eukaryote	NC_007282
Trypansomabrucei TREU927 genome 11 scaffold 2	eukaryote	NT_165287
Acanthamoeba polyphaga mimivirus	virus	NC_006450
Adoxophyes honmai nucleopolyhedrovirus	virus	NC_004690
Aeromonas phage 31	virus	NC_007022
African swine fever virus	virus	NC_001659
Agrotis segetum granulovirus	virus	NC_005839
Alcelaphine herpesvirus 1	virus	NC_002531
Ambystoma tigrinum virus	virus	NC_005832
Amsacta moorei entomopoxvirus	virus	NC_002520
Ateline herpesvirus 3	virus	NC_001987
Autographa californica nucleopolyhedrovirus	virus	NC_001623
bacteriophage 44 RR2.8t	virus	NC_005135
bacteriophage Aeh1	virus	NC_005260
bacteriophage G1	virus	NC_007066
bacteriophage KVP40	virus	NC_005083
bacteriophage RM378	virus	NC_004735
bacteriophage SPBc2	virus	NC_001884
bacteriophage S-PM2 virion	virus	NC_006820
bacteriophage T5 virion	virus	NC_005859
Bombyx mori nucleopolyhedrovirus	virus	NC_001962
Bovine herpesvirus 1	virus	NC_001847
Bovine herpesvirus 4	virus	NC_002665
Bovine herpesvirus 5	virus	NC_005261
Bovine papular stomatitis virus	virus	NC_005337
Callitrichine herpesvirus 3	virus	NC_004367
Camelpoxvirus	virus	NC_003391
Canarypoxvirus	virus	NC_005309
Cercopithecine herpesvirus 1	virus	NC_004812
Cercopithecine herpesvirus 15	virus	NC_006146
Cercopithecine herpesvirus 17	virus	NC_003401
Cercopithecine herpesvirus 2	virus	NC_006560
Cercopithecine herpesvirus 7	virus	NC_002686
Cercopithecine herpesvirus 8	virus	NC_006150
Chimpanzee cytomegalovirus	virus	NC_003521
Choristoneura fumiferana defective nucleopolyhedrovirus	virus	NC_005137
Choristoneura fumiferana MNPV	virus	NC_004778
Chrysodeixis chalcites nucleopolyhedrovirus	virus	NC_007151
Cowpox virus	virus	NC_003663
Cryptophlebia leucotreta granulovirus	virus	NC_005068
Culex nigripalpus baculovirus	virus	NC_003084
Cyanophage P-SSM2	virus	NC_006883
Cyanophage P-SSM4	virus	NC_006884
Cydia pomonella granulovirus	virus	NC_002816
Ectocarpus siliculosus virus	virus	NC_002687
Ectromelia virus	virus	NC_004105
Emiliania huxleyi virus 86	virus	NC_007346
Enterobacteria phage RB43	virus	NC_007023
Enterobacteria phage RB49	virus	NC_005066
Enterobacteria phage RB69	virus	NC_004928
Enterobacteria phage T4	virus	NC_000866
Epiphyas postvittana nucleopolyhedrovirus	virus	NC_003083
Equid herpesvirus 1	virus	NC_001491
Equid herpesvirus 2	virus	NC_001650
Equid herpesvirus 4	virus	NC_001844
Fowlpox virus	virus	NC_002188
Frogvirus 3	virus	NC_005946
Gallid herpesvirus 1	virus	NC_006623
Gallid herpesvirus 2	virus	NC_002229
Gallid herpesvirus 3	virus	NC_002577
Goatpox virus	virus	NC_004003
Helicoverpa armigera nuclearpolyhedrosisvirus	virus	NC_003094
Helicoverpa zea single nucleocapsid nucleopolyhedrovirus	virus	NC_003349
Heliocoverpa armigera nucleopolyhedrovirus G4	virus	NC_002654
Heliothis zea virus 1	virus	NC_004156
Human herpesvirus 1	virus	NC_001806
Human herpesvirus 2	virus	NC_001798
Human herpesvirus 3 (strain Dumas)	virus	NC_001348
Human herpesvirus 4	virus	NC_001345
Human herpesvirus 5 (laboratory strain AD169)	virus	NC_001347
Human herpesvirus 5(wildtype strain Merlin)	virus	NC_006273
Human herpesvirus 6	virus	NC_001664
Human herpesvirus 6B	virus	NC_000898
Human herpesvirus 7	virus	NC_001716
Human herpesvirus 8, genome	virus	NC_003409
Ictalurid herpesvirus 1	virus	NC_001493
Infectious spleen and kidney necrosis virus	virus	NC_003494
Invertebrate iridescent virus 6	virus	NC_003038
Lactobacillus plantarum bacteriophage LP65virion	virus	NC_006565
Lumpy skin disease virus	virus	NC_003027
Lymantria dispar nucleopolyhedrovirus	virus	NC_001973
Lymphocystis disease virus 1	virus	NC_001824
Lymphocystis disease virus-isolate China	virus	NC_005902
Macaca fuscata rhadinovirus	virus	NC_007016
Mamestra configurata NPV-A	virus	NC_003529
Mamestra configurata nucleopolyhedrovirus B	virus	NC_004117
Melanoplus sanguinipes entomopoxvirus	virus	NC_001993
Meleagrid herpesvirus 1	virus	NC_002641
Molluscum contagiosum virus	virus	NC_001731
Monkeypox virus	virus	NC_003310
Muledeerpox virus	virus	NC_006966
Murid herpesvirus 1	virus	NC_004065
Murid herpesvirus 2	virus	NC_002512
Murid herpesvirus 4	virus	NC_001826
Mycobacteriophage Bxz1 virion	virus	NC_004687
Mycobacteriophage Omega virion	virus	NC_004688
Myxoma virus	virus	NC_001132
Orf virus	virus	NC_005336
Orgyia pseudotsugata multicapsid nucleopolyhedrovirus	virus	NC_001875
Ostreid herpesvirus 1	virus	NC_005881
Paramecium bursaria Chlorellavirus 1	virus	NC_000852
Phthorimaea operculella granulovirus	virus	NC_004062
Plutella xylostella granulovirus	virus	NC_002593
Psittacid herpesvirus 1	virus	NC_005264
Psuedomonas phage phiKZ	virus	NC_004629
Rabbit fibroma virus	virus	NC_001266
Rachiplusia ou multiple nucleopolyhedrovirus	virus	NC_004323
Saimiriine herpesvirus 2	virus	NC_001350
Sheeppox virus	virus	NC_004002
Shrimp whitespot syndrome virus	virus	NC_003225
Singapore grouper iridovirus	virus	NC_006549
Spodoptera exigua nucleopolyhedrovirus	virus	NC_002169
Spodoptera litura nucleopolyhedrovirus	virus	NC_003102
Staphylococcus phage K virion	virus	NC_005880
Staphylococcus phage Twort	virus	NC_007021
Suid herpesvirus 1	virus	NC_006151
Swinepox virus	virus	NC_003389
Trichoplusia ni SNPV virus	virus	NC_007383
Tupaia herpesvirus	virus	NC_002794
Vaccinia virus	virus	NC_001559
Variola virus	virus	NC_001611
Xestiac-nigrum granulovirus	virus	NC_002331
Yaba monkey tumorvirus	virus	NC_005179
Yaba-like disease virus	virus	NC_002642

**Table 3.** Viral genomes used for the SOM covering a wide range of viruses, shown in Figure 2. 579 viral genomes have at least 10kb of sequence. This is approximately 35% of all fully sequenced viral genomes available at the time of the analysis.

**Name**	**Accession**
Bovine adenovirus 2	AC_000001
Bovine adenovirus 3	AC_000002
Canine adenovirus type 1	AC_000003
Duck adenovirus 1	AC_000004
Human adenovirus type 12	AC_000005
Human adenovirus type 17	AC_000006
Human adenovirus type 2	AC_000007
Human adenovirus type 5	AC_000008
Porcine adenovirus 5	AC_000009
Simian adenovirus 21	AC_000010
Simian adenovirus 25	AC_000011
Murine adenovirus 1	AC_000012
Fowl adenovirus 9	AC_000013
Fowl adenovirus 1	AC_000014
Human adenovirus type 11	AC_000015
Turkey adenovirus 3	AC_000016
Human adenovirus type 1	AC_000017
Human adenovirus type 7	AC_000018
Human adenovirus type 35	AC_000019
Canine adenovirus type 2	AC_000020
Paramecium bursaria Chlorella virus 1	NC_000852
Viral hemorrhagic septicemia virus	NC_000855
Enterobacteria phage T4	NC_000866
Alteromonas phage PM2	NC_000867
Streptococcus thermophilus bacteriophage Sfi19	NC_000871
Streptococcus thermophilus bacteriophage Sfi21	NC_000872
Lactobacillus bacteriophage phi adh	NC_000896
Human herpesvirus 6B	NC_000898
Fowl adenovirus D	NC_000899
Bacteriophage VT2-Sa	NC_000902
Snakehead rhabdovirus	NC_000903
Bacteriophage 933W	NC_000924
Enterobacteria phage Mu	NC_000929
Acyrthosiphon pisum bacteriophage APSE-1	NC_000935
Murine adenovirus A	NC_000942
Murray Valley encephalitis virus	NC_000943
Myxomavirus	NC_001132
Rabbit fibromavirus	NC_001266
Bacteriophage phi YeO3-12	NC_001271
Enterobacteria phage 186	NC_001317
Mycobacterium phage L5	NC_001335
Sulfolobus spindle-shaped virus 1	NC_001338
Human herpesvirus 4	NC_001345
Human herpesvirus 5 (laboratory strain AD169)	NC_001347
Human herpesvirus 3 (strain Dumas)	NC_001348
Saimiriine herpesvirus 2	NC_001350
Simian foamy virus	NC_001364
Human adenovirus C	NC_001405
Bacteriophage lambda	NC_001416
Enterobacteria phage PRD1	NC_001421
Bacillus phage PZA	NC_001423
Japanese encephalitis virus	NC_001437
Achole plasmaphage L2	NC_001447
Venezuelan equine encephalitis virus	NC_001449
Avian infectious bronchitis virus	NC_001451
Human adenovirus F	NC_001454
Human adenovirus A	NC_001460
Bovine viral diarrheavirus 1	NC_001461
Dengue virus type 2	NC_001474
Dengue virus type 3	NC_001475
Dengue virus type 1	NC_001477
Equid herpesvirus 1	NC_001491
Cryphonectria hypovirus 1	NC_001492
Ictalurid herpesvirus 1	NC_001493
Measles virus	NC_001498
O’nyong-nyong virus	NC_001512
Rabies virus	NC_001542
Ross River virus	NC_001544
Sindbis virus	NC_001547
Sendai virus	NC_001552
Vaccinia virus	NC_001559
Vesicular stomatitis Indiana virus	NC_001560
West Nile virus	NC_001563
Cell fusing agent virus	NC_001564
Beet yellows virus	NC_001598
Enterobacteria phage T7	NC_001604
Lake Victoria marburg virus	NC_001608
Bacteriophage P4	NC_001609
Variola virus	NC_001611
Sonchus yellow net virus	NC_001615
Autographa californica nucleopolyhedrovirus	NC_001623
Rice tungro spherical virus	NC_001632
Equid herpesvirus 2	NC_001650
Infectious hematopoietic necrosis virus	NC_001652
African swine fever virus	NC_001659
Citrus tristeza virus	NC_001661
Human herpesvirus 6	NC_001664
Tick-borne encephalitis virus	NC_001672
Haemophilus phage HP1	NC_001697
Lactococcus phage c2	NC_001706
Human herpesvirus 7	NC_001716
Fowl adenovirus A	NC_001720
Human immunodeficiency virus 2	NC_001722
Snakehead retrovirus	NC_001724
Molluscum contagiosum virus	NC_001731
Canine adenovirus	NC_001734
Human foamy virus	NC_001736
Human respiratory syncytial virus	NC_001781
Papaya ringspot virus	NC_001785
Barmah Forest virus	NC_001786
Human spuma retrovirus	NC_001795
Human parainfluenza virus 3	NC_001796
Human herpesvirus 2	NC_001798
Respiratory syncytial virus	NC_001803
Human herpesvirus 1	NC_001806
Louping ill virus	NC_001809
Duck adenovirus A	NC_001813
Lymphocystis disease virus 1	NC_001824
Streptococcus phage Cp-1	NC_001825
Murid herpesvirus 4	NC_001826
Bovine foamy virus	NC_001831
Bacteriophage sk1	NC_001835
Little cherry virus 1	NC_001836
Sweet potato feathery mottle virus	NC_001841
Equid herpesvirus 4	NC_001844
Murine hepatitis virus strain A59	NC_001846
Bovine herpesvirus 1	NC_001847
Walleye dermal sarcoma virus	NC_001867
Simian-Human immunodeficiency virus	NC_001870
Feline foamy virus	NC_001871
Rhopalosiphum padi virus	NC_001874
Orgyia pseudotsugata nucleopolyhedrovirus	NC_001875
Bovine adenovirus B	NC_001876
Bacteriophage SPBc2	NC_001884
Enterobacteria phage P2	NC_001895
Mycobacteriophage D29	NC_001900
Bacteriophage N15	NC_001901
Methanobacterium phage psiM2	NC_001902
Hendra virus	NC_001906
Bacteriophage bIL170	NC_001909
Canine distemper virus	NC_001921
Igbo Ora virus	NC_001924
Mycoplasma arthritidis bacteriophage MAV1	NC_001942
Hemorrhagic enteritis virus	NC_001958
Porcine reproductive and respiratory syndrome virus	NC_001961
Bombyx mori nucleopolyhedrovirus	NC_001962
Lymantria dispar nucleopolyhedrovirus	NC_001973
Bacteriophage phi-C31	NC_001978
Ateline herpesvirus 3	NC_001987
Bovine respiratory syncytial virus	NC_001989
Melanoplus sanguinipes entomopox virus	NC_001993
Yellow fever virus	NC_002031
Bovine viral diarrhea virus genotype 2	NC_002032
Human adenovirus D	NC_002067
Streptococcus thermophilus bacteriophage DT1	NC_002072
Bovine parainfluenza virus3	NC_002161
Enterobacteria phage HK022	NC_002166
Bacteriophage HK97	NC_002167
Spodoptera exigua nucleopolyhedrovirus	NC_002169
Streptococcus thermophilus bacteriophage 7201	NC_002185
Fowlpox virus	NC_002188
Tupaia paramyxovirus	NC_002199
Mumps virus	NC_002200
Equine foamy virus	NC_002201
Streptococcus thermophilus bacteriophage Sfi11	NC_002214
Gallid herpesvirus 2	NC_002229
Northern cereal mosaic virus	NC_002251
Transmissible gastroenteritis virus	NC_002306
Staphylococcus aureus bacteriophage PVL	NC_002321
Xestiac-nigrum granulovirus	NC_002331
Enterobacteria phage P22	NC_002371
Pseudomonas phage D3	NC_002484
Staphylococcus aureus prophage phiPV83	NC_002486
Frog adenovirus	NC_002501
Murid herpesvirus 2	NC_002512
Ovine adenovirus A	NC_002513
Mycoplasma virus P1	NC_002515
Roseophage SIO1	NC_002519
Amsacta moorei entomopox virus	NC_002520
Bovine ephemeral fever virus	NC_002526
Alcelaphine herpesvirus 1	NC_002531
Equine arteritis virus	NC_002532
Lactate dehydrogenase-elevating virus	NC_002534
Zaire ebola virus	NC_002549
Gallid herpesvirus 3	NC_002577
Plutella xylostella granulovirus	NC_002593
Newcastle disease virus	NC_002617
Methanothermobacter wolfeii prophage psiM100	NC_002628
Dengue virus type 4	NC_002640
Meleagrid herpesvirus 1	NC_002641
Yaba-like disease virus	NC_002642
Human coronavirus 229E	NC_002645
Bacillus phage GA-1	NC_002649
Heliocoverpa armigera nucleopolyhedrovirus G4	NC_002654
Mycobacteriophage Bxb1	NC_002656
Classical swine fever virus	NC_002657
Staphylococcus aureus temperate phage phi SLT	NC_002661
Bovine herpesvirus 4	NC_002665
Bacteriophage bIL285	NC_002666
Bacteriophage bIL286	NC_002667
Bacteriophage bIL309	NC_002668
Bacteriophage bIL310	NC_002669
Bacteriophage bIL311	NC_002670
Bacteriophage bIL312	NC_002671
Bovine adenovirus D	NC_002685
Cercopithecine herpesvirus 7	NC_002686
Ectocarpus siliculosus virus	NC_002687
Porcine adenovirus C	NC_002702
Bacteriophage Tuc2009	NC_002703
Nipah virus	NC_002728
Bacteriophage HK620	NC_002730
Lactococcus lactis bacteriophage TP901-1	NC_002747
Tupaia herpesvirus	NC_002794
Lactococcus phage BK5-T	NC_002796
Spring viremia of carp virus	NC_002803
Cydia pomonella granulovirus	NC_002816
Taura syndrome virus	NC_003005
Lumpy skin disease virus	NC_003027
Invertebrate iridescent virus 6	NC_003038
Avian paramyxovirus 6	NC_003043
Bovine coronavirus	NC_003045
Streptococcus pneumoniae bacteriophage MM1	NC_003050
Epiphyas postvittana nucleopolyhedrovirus	NC_003083
Culex nigripalpus baculovirus	NC_003084
Bacteriophage Mx8	NC_003085
Simian hemorrhagic fever virus	NC_003092
Helicoverpa armigera nuclearpolyhedrosis virus	NC_003094
Spodopteralitura nucleopolyhedrovirus	NC_003102
Temperate phage PhiNIH1.1	NC_003157
Sulfolobus islandicus filamentous virus	NC_003214
Semliki forest virus	NC_003215
Bacteriophage A118	NC_003216
Shrimp white spot syndrome virus	NC_003225
Australian bat lyssa virus	NC_003243
Human adenovirus E	NC_003266
Bacteriophage phiCTX	NC_003278
Bacteriophage phiETA	NC_003288
Bacteriophage PSA	NC_003291
Bacteriophage T3	NC_003298
Bacteriophage phiE125	NC_003309
Monkeypox virus	NC_003310
Bacteriophage K139	NC_003313
Haemophilus phage HP2	NC_003315
Sinorhizobium meliloti phage PBC5	NC_003324
Halovirus HF2	NC_003345
Helicoverpa zea nucleopolyhedrovirus	NC_003349
Bacteriophage P27	NC_003356
Mycobacteriophage TM4	NC_003387
Swinepox virus	NC_003389
Cyanophage P60	NC_003390
Camelpox virus	NC_003391
Cercopithecine herpesvirus 17	NC_003401
Human herpesvirus 8	NC_003409
Mayaro virus	NC_003417
Sleeping disease virus	NC_003433
Porcine epidemic diarrhea virus	NC_003436
Human parainfluenza virus 2	NC_003443
Shigella flexneri bacteriophage V	NC_003444
Human parainfluenza virus 1 strain Washington/1964	NC_003461
Infectious spleen and kidney necrosis virus	NC_003494
Chimpanzee cytomegalovirus	NC_003521
Bacteriophage phi3626	NC_003524
Stx2 converting bacteriophage I	NC_003525
Mamestra configurata NPV-A	NC_003529
Cryphonectria hypovirus	NC_003534
Dasheen mosaic virus	NC_003537
Lettuce mosaic virus	NC_003605
Maize chlorotic dwarf virus	NC_003626
Modoc virus	NC_003635
Cowpox virus	NC_003663
Rio Bravo virus	NC_003675
Apoi virus	NC_003676
Pestivirus Reindeer-1	NC_003677
Pestivirus Giraffe-1	NC_003678
Border disease virus 1	NC_003679
Powassan virus	NC_003687
Langat virus	NC_003690
Rice yellow stunt virus	NC_003746
Acyrthosiphon pisum virus	NC_003780
Sweet potato mild mottle virus	NC_003797
Eastern equine encephalitis virus	NC_003899
Aura virus	NC_003900
Vibriophage VpV262	NC_003907
Western equine encephalomyelitis virus	NC_003908
Salmon pancreas disease virus	NC_003930
Tamana bat virus	NC_003996
Human adenovirus B	NC_004001
Sheeppox virus	NC_004002
Goatpox virus	NC_004003
Leek yellow stripe virus	NC_004011
Ovine adenovirus 7	NC_004037
Phthorimaea operculella granulovirus	NC_004062
Murid herpesvirus 1	NC_004065
Lactococcus lactisbacteriophageul36	NC_004066
Tiomanvirus	NC_004074
VirusPhiCh1	NC_004084
Sulfolobus islandicus rod-shaped virus 2	NC_004086
Sulfolobus islandicus rod-shaped virus 1	NC_004087
Ectromelia virus	NC_004105
Lactobacillus casei bacteriophage A2	NC_004112
Mamestra configurata nucleopolyhedrovirus B	NC_004117
Montana myotis leukoencephalitis virus	NC_004119
Human metapneumovirus	NC_004148
Heliothis zea virus 1	NC_004156
Dugbe virus segment L	NC_004159
Reston Ebola virus	NC_004161
Chikungunya virus	NC_004162
Bacteriophage B103	NC_004165
Bacteriophage SPP1	NC_004166
Bacteriophage phi-105	NC_004167
Bacteriophage r1t	NC_004302
Streptococcus thermophilus bacteriophage O1205	NC_004303
Bacteriophage phig1e	NC_004305
Salmonella typhimurium phage ST64B	NC_004313
Rachiplusia ou multiple nucleopolyhedrovirus	NC_004323
Burkholderia cepacia phage Bcep781	NC_004333
Salmonella typhimurium bacteriophage ST64T	NC_004348
Alkhurma virus	NC_004355
Callitrichine herpesvirus 3	NC_004367
Treeshrew adenovirus	NC_004453
Vibrio harveyi bacteriophage VHML	NC_004456
Bacteriophage IN93	NC_004462
Pseudomonas aeruginosa phage PaP3	NC_004466
Streptococcus pyogenes phage 315.1	NC_004584
Streptococcus pyogenes phage 315.2	NC_004585
Streptococcus pyogenes phage 315.3	NC_004586
Streptococcus pyogenes phage 315.4	NC_004587
Streptococcus pyogenes phage 315.5	NC_004588
Streptococcus pyogenes phage 315.6	NC_004589
Staphylococcus aureus phage phi11	NC_004615
Staphylococcus aureus phage phi12	NC_004616
Staphylococcus aureus phage phi13	NC_004617
Pseudomonas phage phiKZ	NC_004629
Bacteriophage phi-BT1	NC_004664
Pseudomonas phage gh-1	NC_004665
Grapevine leaf roll-associated virus 3	NC_004667
Staphylococcus phage 44AHJD	NC_004678
Staphylococcus aureus phage phiP68	NC_004679
Mycobacteriophage Che8	NC_004680
Mycobacteriophage CJW1	NC_004681
Mycobacteriophage Bxz2	NC_004682
Mycobacteriophage Che9c	NC_004683
Mycobacteriophage Rosebush	NC_004684
Mycobacteriophage Corndog	NC_004685
Mycobacteriophage Che9d	NC_004686
Mycobacteriophage Bxz1	NC_004687
Mycobacteriophage Omega	NC_004688
Mycobacteriophage Barnyard	NC_004689
Adoxophyes honmai nucleopolyhedrovirus	NC_004690
SARS coronavirus	NC_004718
Grapevine rootstock stem lesion associated virus	NC_004724
Bacteriophage RM378	NC_004735
Staphylococcus phage phiN315	NC_004740
Bacteriophage L-413C	NC_004745
Lactococcus phage P335	NC_004746
Enterobacteria phage epsilon15	NC_004775
Yersinia pestis phage phiA1122	NC_004777
Choristoneura fumiferana MNPV	NC_004778
Cercopithecine herpesvirus 1	NC_004812
Phage phi4795	NC_004813
Streptococcus phage C1	NC_004814
Bacteriophage phBC6A51	NC_004820
Bacteriophage phBC6A52	NC_004821
Bacteriophage Aaphi23	NC_004827
Deformed wing virus	NC_004830
Enterobacteria phage SP6	NC_004831
Xanthomonas oryzae bacteriophage Xp10	NC_004902
Stx1 converting bacteriophage	NC_004913
Stx2 converting bacteriophage II	NC_004914
Halovirus HF1	NC_004927
Enterobacteria phage RB69	NC_004928
Streptococcus mitis phage SM1	NC_004996
Papaya leaf-distortion mosaic potyvirus	NC_005028
Onion yellow dwarf virus	NC_005029
Goose paramyxovirus SF02	NC_005036
Adoxophyes orana granulovirus	NC_005038
Yokose virus	NC_005039
Bacteriophage phiKMV	NC_005045
Bacteriophage WPhi	NC_005056
Omsk hemorrhagic fever virus	NC_005062
Kamiti River virus	NC_005064
Little cherry virus 2	NC_005065
Enterobacteria phage RB49	NC_005066
Cryptophlebia leucotreta granulovirus	NC_005068
Bacteriophage PY54	NC_005069
Bacteriophage KVP40	NC_005083
Fer-de-lance virus	NC_005084
Burkholderia cepacia phage BcepNazgul	NC_005091
Hirame rhabdovirus	NC_005093
Bacteriophage 44RR2.8t	NC_005135
Choristoneura fumiferana nucleopolyhedrovirus	NC_005137
Human coronavirus OC43	NC_005147
Bacteriophage D3112	NC_005178
Yaba monkey tumor virus	NC_005179
Bacillus thuringiensis bacteriophage Bam35c	NC_005258
Mycobacteriophage PG1	NC_005259
Bacteriophage Aeh1	NC_005260
Bovine herpesvirus 5	NC_005261
Burkholderia cepacia phage Bcep22	NC_005262
Burkholderia cenocepacia phage Bcep1	NC_005263
Psittacid herpesvirus 1	NC_005264
Sulfolobus spindle-shaped virus 2	NC_005265
Bacteriophage Felix01	NC_005282
Dolphin morbillivirus	NC_005283
Bacteriophage phi1026b	NC_005284
Bacteriophage EJ-1	NC_005294
Crimean-Congo hemorrhagic fever virus segment L	NC_005301
Canarypox virus	NC_005309
Orfvirus	NC_005336
Bovine papularstomatitis virus	NC_005337
Mossman virus	NC_005339
Bacteriophage PSP3	NC_005340
Burkholderia cepacia phage Bcep43	NC_005342
Enterobacteria phage Sf6	NC_005344
Bacteriophage VWB	NC_005345
Lactobacillus johnsonii prophage Lj928	NC_005354
Lactobacillus johnsonii prophage Lj965	NC_005355
Bacteriophage 77	NC_005356
Bordetella phage BPP-1	NC_005357
Sulfolobus spindle-shaped virus Ragged Hills	NC_005360
Sulfolobus spindle-shaped virus Kamchatka-1	NC_005361
Bordetella phage BMP-1	NC_005808
Bordetella phage BIP-1	NC_005809
Bacteriophage phiLC3	NC_005822
Acidianus filamentus virus 1	NC_005830
Human coronavirus NL63	NC_005831
Ambystoma tigrinum virus	NC_005832
Enterobacteria phage T1	NC_005833
Agrotis segetum granulovirus	NC_005839
Salmonella typhimurium bacteriophage ST104	NC_005841
Enterobacteria phage P1	NC_005856
Bacteriophage phiKO2	NC_005857
Bacteriophage T5	NC_005859
Porcine adenovirus A	NC_005869
Pyrobaculum spherical virus	NC_005872
Kakugo virus	NC_005876
Vibriophage VP2	NC_005879
Staphylococcus phage K	NC_005880
Ostreid herpesvirus 1	NC_005881
Burkholderia cenocepacia phage BcepMu	NC_005882
Pseudomonas aeruginosa bacteriophage PaP2	NC_005884
Actinoplanes phage phiAsp2	NC_005885
Burkholderia cenocepacia phage BcepB1A	NC_005886
Burkholderia cepacia complex phage BcepC6B	NC_005887
Vibriophage VP5	NC_005891
Sulfolobus turreted icosahedral virus	NC_005892
Bacteriophage phiAT3	NC_005893
Lymphocystis disease virus-isolate China	NC_005902
Neodiprion sertifer nucleopolyhedrovirus	NC_005905
Neodiprion lecontei NPV	NC_005906
Frog virus 3	NC_005946
Bacteriophage phiMFV1	NC_005964
Maize fine streak virus	NC_005974
Maize mosaic virus	NC_005975
Simian adenovirus A	NC_006144
Cercopithecine herpesvirus 15	NC_006146
Cercopithecine herpesvirus 8	NC_006150
Suid herpesvirus 1	NC_006151
Watermelon mosaic virus	NC_006262
Sulfolobus tengchongensis spindle-shaped virus STSV1	NC_006268
Human herpesvirus 5 (wildtype strain Merlin)	NC_006273
Rinderpest virus	NC_006296
Bovine adenovirus A	NC_006324
Bacteriophage 11b	NC_006356
Peste-des-petits-ruminants virus	NC_006383
Simian parainfluenza virus 41	NC_006428
Mokola virus	NC_006429
Simian parainfluenza virus5	NC_006430
Sudan ebola virus	NC_006432
Acanthamoeba polyphaga mimivirus	NC_006450
Varroa destructor virus 1	NC_006494
Bacteriophage B3	NC_006548
Singapore grouper iridovirus	NC_006549
Usutu virus	NC_006551
Pseudomonas aeruginosa phage F116	NC_006552
Thermoproteus tenax spherical virus 1	NC_006556
Bacillus clarkii bacteriophage BCJA1c	NC_006557
Getah virus	NC_006558
Cercopithecine herpesvirus 2	NC_006560
Lactobacillus plantarum bacteriophage LP65	NC_006565
Human coronavirus HKU1	NC_006577
Pneumonia virus of mice J3666	NC_006579
Gallid herpesvirus 1	NC_006623
Cotesia congregata virus segment Circle 1	NC_006633
Cotesia congregata virus segment Circle 2	NC_006634
Cotesia congregata virus segment Circle 3	NC_006635
Cotesia congregata virus segment Circle 4	NC_006636
Cotesia congregata virus segment Circle 5	NC_006637
Cotesia congregata virus segment Circle 6	NC_006638
Cotesia congregata virus segment Circle 7	NC_006639
Cotesia congregata virus segment Circle 9	NC_006641
Cotesia congregata virus segment Circle 10	NC_006642
Cotesia congregata virus segment Circle 11	NC_006643
Cotesia congregata virus segment Circle 12	NC_006644
Cotesia congregata virus segment Circle 13	NC_006645
Cotesia congregata virus segment Circle 14	NC_006646
Cotesia congregata virus segment Circle 17	NC_006648
Cotesia congregata virus segment Circle 18	NC_006649
Cotesia congregata virus segment Circle 19	NC_006650
Cotesia congregata virus segment Circle 20	NC_006651
Cotesia congregata virus segment Circle 22	NC_006653
Cotesia congregata virus segment Circle 23	NC_006654
Cotesia congregata virus segment Circle 25	NC_006655
Cotesia congregata virus segment Circle 26	NC_006656
Cotesia congregata virus segment Circle 30	NC_006657
Cotesia congregata virus segment Circle 31	NC_006658
Cotesia congregata virus segment Circle 32	NC_006659
Cotesia congregata virus segment Circle 33	NC_006660
Cotesia congregata virus segment Circle 35	NC_006661
Cotesia congregata virus segment Circle 36	NC_006662
Bacteriophage S-PM2	NC_006820
Murine hepatitis virus strain JHM	NC_006852
Simian adenovirus 1	NC_006879
Cyanophage P-SSP7	NC_006882
Cyanophage P-SSM2	NC_006883
Cyanophage P-SSM4	NC_006884
Lactobacillus plantarum bacteriophage phiJL-1	NC_006936
Bacteriophage phiJL001	NC_006938
Bacteriophage KS7	NC_006940
Taro vein chlorosis virus	NC_006942
Mint virus 1	NC_006944
Bacillus thuringiensis phage GIL16c	NC_006945
Karshi virus	NC_006947
Salmonella typhimurium bacteriophage ES18	NC_006949
Listonella pelagia phage phiHSIC	NC_006953
Muledeerpox virus	NC_006966
Vaccinia virus	NC_006998
Macaca fuscata rhadinovirus	NC_007016
Streptococcus thermophilus bacteriophage 2972	NC_007019
Tupaia rhabdovirus	NC_007020
Staphylococcus phage Twort	NC_007021
Aeromonas phage 31	NC_007022
Enterobacteria phage RB43	NC_007023
Xanthomonas campestris pv. pelargonii phage Xp15	NC_007024
Feline coronavirus	NC_007025
Microplitis demolitor bracovirus segment G	NC_007034
Microplitis demolitor bracovirus segment H	NC_007035
Microplitis demolitor bracovirus segment J	NC_007036
Microplitis demolitor bracovirus segment K	NC_007037
Microplitis demolitor bracovirus segment M	NC_007038
Microplitis demolitor bracovirus segment N	NC_007039
Microplitis demolitor bracovirus segment L	NC_007040
Microplitis demolitor bracovirus segment I	NC_007041
Microplitis demolitor bracovirus segment O	NC_007044
Bacteriophage PT1028	NC_007045
Bacteriophage 66	NC_007046
Bacteriophage 187	NC_007047
Bacteriophage 69	NC_007048
Bacteriophage 53	NC_007049
Bacteriophage 85	NC_007050
Bacteriophage 2638A	NC_007051
Bacteriophage 42e	NC_007052
Bacteriophage 3A	NC_007053
Bacteriophage 47	NC_007054
Bacteriophage 37	NC_007055
Bacteriophage EW	NC_007056
Bacteriophage 96	NC_007057
Bacteriophage ROSA	NC_007058
Bacteriophage 71	NC_007059
Bacteriophage 55	NC_007060
Bacteriophage 29	NC_007061
Bacteriophage 52A	NC_007062
Bacteriophage 88	NC_007063
Bacteriophage 92	NC_007064
Bacteriophage X2	NC_007065
Bacteriophage G1	NC_007066
Phytophthora endorna virus 1	NC_007069
Burkholderia pseudomallei phage phi52237	NC_007145
Vibriophage VP4	NC_007149
Chrysodeixis chalcites nucleopolyhedrovirus	NC_007151
Bacteriophage SH1	NC_007217
Bacteriophage JK06	NC_007291
Emiliania huxleyi virus 86	NC_007346
Trichoplusia ni SNPV virus	NC_007383
Acidianus two-tailed virus	NC_007409
Shallot yellow stripe virus	NC_007433
Breda virus	NC_007447
Grapevine leaf roll-associated virus 2	NC_007448
Enterobacteria phage L17	NC_007449
Enterobacteria phage PR3	NC_007450
Enterobacteria phage PR4	NC_007451
Enterobacteria phage PR5	NC_007452
Enterobacteria phage PR772	NC_007453
J-virus	NC_007454
Coliphage K1F	NC_007456
Bacillus anthracis phage Cherry	NC_007457
Bacillus anthracis phage Gamma	NC_007458
Burkholderia cepacia phage Bcep176	NC_007497
Bacteriophage Lc-Nu	NC_007501

## Footnotes

† Both *genome signature* and *genomic signature* are used interchangeably in the field, including by their originators. However, the term *genome* signature is to be preferred, since *genomic* signature is used in the field of molecular diagnostics to refer to a genotype correlated with medical symptoms or prognosis (e.g. Russo *et al.* 2005)
